# Dual Isotopologue Profiling of Bacterial Pathogens and their Host Cells: Metabolic Adaptation of Human Macrophages and Fallopian Tube Cells to Intracellular *Chlamydia trachomatis*

**DOI:** 10.1021/acschembio.5c00268

**Published:** 2025-07-28

**Authors:** Sandra Radziej, Adriana Moldovan, Mark Klöpfer, Werner Goebel, Thomas Rudel, Wolfgang Eisenreich

**Affiliations:** † 9184 Technische Universität München, Bavarian NMR Center − Structural Membrane Biochemistry, Department of Chemistry, Lichtenbergstraße 4, D-85747 Garching, Germany; ‡ Julius-Maximilian-Universität Würzburg, Chair of Microbiology, Am Hubland, D-97074 Würzburg, Germany

## Abstract

*Chlamydia trachomatis* is a Gram-negative bacterium that utilizes multiple host-derived substrates to ensure its intracellular survival. In this study, human fallopian tube (HFT) cells, and human macrophages polarized toward a pro-inflammatory (M1-like) or anti-inflammatory (M2-like) state were infected with *C. trachomatis* and cocultivated in the presence of [U-^13^C_6_]­glucose. Samples were analyzed *in toto* by dual isotopologue profiling with a focus on specific bacterial and host-specific metabolites. Immunofluorescence and ultrastructural analysis, as well as detection of the bacteria-specific metabolites (i.e., the branched-chain iso-C15:0 and anteiso-C15:0 fatty acids, and the cell wall component meso-diaminopimelic acid), confirmed that HFT cells and M2-like, but not M1-like macrophages, allow replication of *C. trachomatis*. The ^13^C-labeling patterns in these metabolites reflected their known biosynthetic pathways, but also upstream carbon fluxes via the uptake of host amino acids and glucose phosphate into the intracellular bacteria. Differential analysis of infected vs noninfected host cells showed that, in HFT cells and M2-like macrophages, the chlamydial infection upregulated glucose uptake into the host cells, glucose conversion into pyruvate and lactate via host glycolysis, and release of lactate into the medium. The rates of these processes were higher in HFT cells than in M2-like macrophages. We here establish dual isotopologue profiling as a suitable method to analyze the dynamics of host-intracellular pathogen interactions.

## Graphical abstract

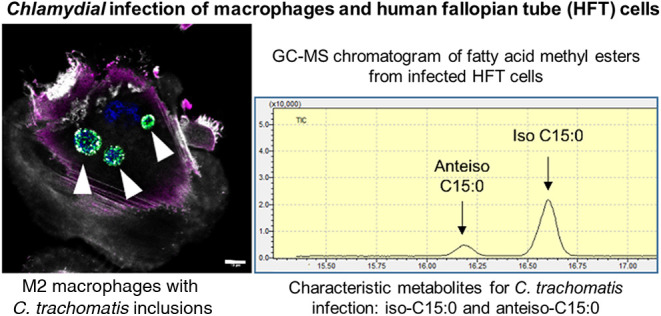

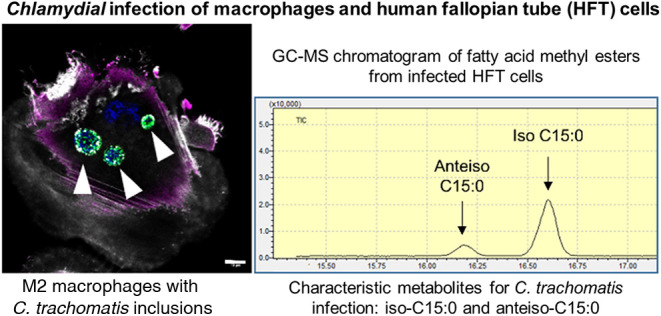

## Introduction

The human pathogen *Chlamydia trachomatis* (*C. trachomatis*) is a Gram-negative, obligate intracellular bacterium which causes trachoma, genital tract and rectum infections.^1^ Its life cycle is characterized by two morphologically different states: the infectious, nonreplicative elementary body (EB) and the noninfectious, yet replicative, reticulate body (RB). To initiate infection, EBs bind onto the host cells and, following entry, a pathogen-containing vacuole termed “*inclusion*” is formed, where EBs convert into RBs and start replication. After reconverting into EBs, the bacteria are extruded or released from the host cells and potentially infect new ones.^2^ Under stress conditions (e.g., amino acid starvation, treatment with interferon gamma), *C. trachomatis* can convert from the RB form into persistent aberrant reticulate bodies (ARB). In this “persistent state”, the bacteria are viable, replicate their genome, but metabolic activity is decreased and cell division is halted.^3^ This phenotype is reversible and, as soon as stress conditions have ceased, bacteria resume cell division and continue their developmental cycle.^4^

With a genome of only ∼1 Mb,^5^ *C. trachomatis* has a reduced core metabolic network and requires the uptake of multiple metabolites from the host cell. The tricarboxylic acid cycle (TCA cycle) of the pathogen is incomplete, due to the absence of three key enzymes: isocitrate dehydrogenase, aconitase, and citrate synthase. Although substrates like malate or glutamine can directly fill the TCA cycle, survival and growth depend on the supply of multiple host-derived nutrient sources.^6,7^ During infection of cultured host cells, *C. trachomatis* scavenges nucleotides,^8^ amino acids,^9^ lipids,^10,11^ but also glucose 6-phosphate (G6P). After uptake, G6P is converted into pyruvate via glycolysis.^12^ In EBs, ATP is generated from G6P either by substrate phosphorylation via glycolysis or oxidative phosphorylation. RBs, however, mainly scavenge ATP from the host cell.^13^

Due to its fast proliferation rate, the cervical epithelial HeLa cell line is often used to study cell response during infection with *C. trachomatis*. However, other epithelial cell types, both primary and cultured, are suitable models for studying *Chlamydia–*host interactions.^14^ *Chlamydia* readily infects also other cell types, including immune cells.^15^ Subtypes of macrophages of both murine and human origin were shown to serve as a replicative niche for the pathogen.^16–18^ Macrophages are specialized phagocytic cells of the innate immune system, with roles ranging from pathogen control to wound repair.^19^ Due to their unique functional characteristics, several macrophage populations were reported *in vivo*.^19,20^ A conventional approach for studying macrophages *in vitro* is by assigning them a so-called *classically activated* (“M1”) or *alternatively activated* (“M2”) phenotype.^21^ The “M1” phenotype is induced by bacterial lipopolysaccharides (LPS) and cytokines such as interferon gamma (IFN-γ) and is typically associated with pro-inflammatory and antimicrobial responses. In contrast, the anti-inflammatory “M2” phenotype - induced by interleukin 4 (IL-4) or interleukin 13 (IL-13) - promotes cell proliferation and tissue repair.^21,22^

Little is known on how the metabolism of primary host cells is reprogrammed upon infection and in particular which factors from the host cell and intracellular bacteria are involved in this metabolic reprogramming.^23^

By employing ^13^C-flux analysis and isotopologue profiling^24,25^ in different host cell models for *C. trachomatis* infection, the analytical focus of this study is represented by metabolites which are specific either for bacterial or host metabolism, and consequently, the analysis was done *in toto* with mixed fractions of infected host cells, without prior separation. In analogy to dual-RNA-seq technology, we termed this approach “dual-isotopologue profiling”.

## Results and Discussion

In this study, human immortalized fallopian tube (HFT) cells, as well as human blood monocyte-derived classically activated “M1″-like macrophages (GM-CSF/IFN-γ/LPS) and alternatively activated “M2”-like macrophages (M-CSF/IL-4) were used and will be henceforth referred to as M1Φ and M2Φ, respectively.

To better understand how *C. trachomatis* affects the metabolism of primary hosts, we performed labeling experiments with HFT cells as well as human M1Φ or M2Φ using 11 mM [U-^13^C]­glucose. Without prior separation of the cell pellet into host cell and bacteria fractions, respectively, ^13^C-labeling patterns of either host- or bacteria-specific metabolites were detected with GC-MS and NMR and served to reconstruct crucial metabolic fluxes in both bacteria and host cells in response to infection.

Infected and uninfected cells were extracted and hydrolyzed *in toto* for GC-MS analysis and ^13^C-profiles in metabolites released into the culture supernatants were analyzed by NMR spectroscopy. More specifically, equivalents of the infected or noninfected cell pellets were *in toto* (i) hydrolyzed under aqueous harsh acidic conditions to protein-derived amino acids, (ii) extracted using cold methanol to obtain polar metabolites such as intermediates of the TCA cycle, or (iii) hydrolyzed under methanolic mild acidic conditions to obtain lipid-derived fatty acid methyl esters. These mixtures were reacted into appropriate derivatives and then studied by quantitative GC-MS analysis. The molecular weight of a compound containing no ^13^C atoms was referred to with M in this notation. M+1 referred to the same compound containing one ^13^C atom, M+2 two ^13^C atoms etc. Following these protocols, ^13^C-enrichments (mol% ^13^C-excess) and relative isotopologue fractions (i.e., relative % M+1, M+2, M+3, etc. containing one, two, three, etc. ^13^C-atoms, respectively, normalized to a total value of 100 irrespective of their absolute ^13^C-excess) were obtained for each metabolite.

### Data Management and Statistics

Labeling experiments were repeated two or three times (biological replicates) and measured in triplicates (technical replicates). In our analysis, we observed for all metabolites of the technical replicates that the variation of absolute ^13^C-values in equivalent compounds was minor (<1%), whereas the variation of the absolute ^13^C-values was large in equivalent compounds from the biological replicates (>10%). This can be illustrated for protein-derived alanine from the three biological replicates R1–R3 of experiments with HFT cells (Figure 1). Consequently, the uptake of exogenous [U-^13^C_6_]­glucose and the rates of its metabolic conversion into alanine considerably differed in the biological batches.

**Figure 1:**
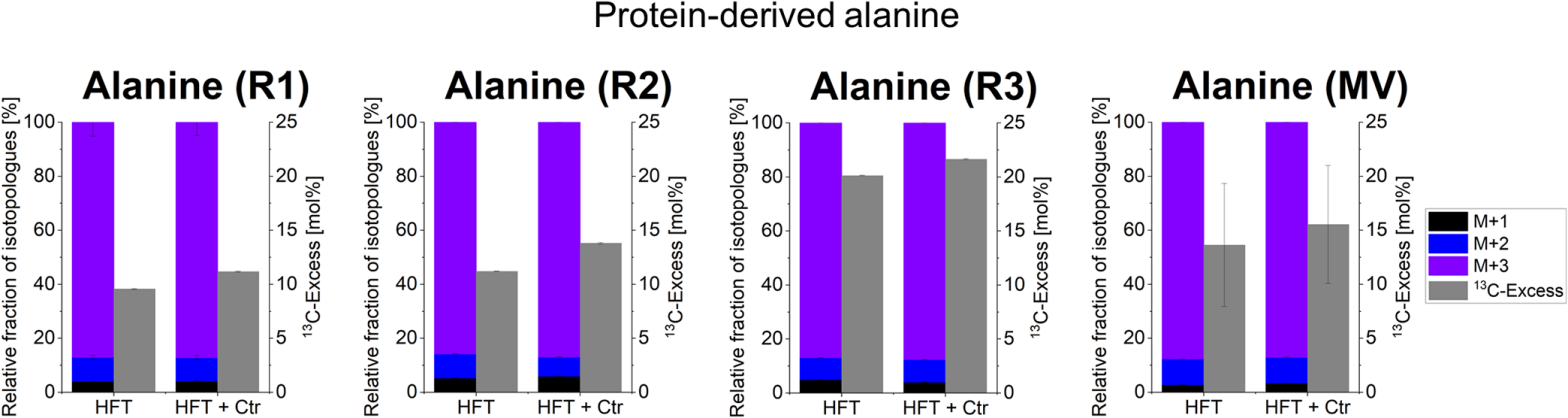
^13^C-Excess in mol% and their isotopologue profiles of the protein-derived amino acid alanine from infected and uninfected HFT cells. M+X represents the molecular mass fraction of alanine specimens carrying X ^13^C-atoms. Error bars show the standard deviations from three separate biological replicates (R1, R2, and R3) with three technical replicates as well as the mean value (MV) from three biological replicates with three technical replicates; (Ctr = *C. trachomatis*).

However, across all biological replicates, the ^13^C-excess values of alanine from the infected cells were increased by a similar factor (i.e., 1.2) in comparison to the noninfected cells from the same batch. The same conclusion can be drawn for other metabolites from corresponding R1–R3 batches, (i.e., corresponding batches of noninfected vs infected HFT cells) and for the experimental replicates with human primary macrophages (see below). However, we attribute this variation to inherent differences in the biological status of the cells used for infection. In the case of human macrophages, variation observed across biological replicates likely stems mainly from the origin of the biological material (i.e., cells were isolated from blood samples from different donors).

In contrast, the relative fractions were almost identical for alanine from noninfected or infected HFT cells from the different batches (Figure 1). Based on these considerations, it follows that the metabolic pathways were highly similar, if not identical in cells from the various batches of biological replicates, but the absolute carbon fluxes were different. The standardized procedures dealing with mean absolute values and substantial mean deviations would therefore introduce an unwanted bias or even obscure the significance of our data sets. Therefore, we will not discuss in the following the mean ^13^C-excess values of metabolites averaged over the biological replicates, but rather refer to a specific batch of cells, i.e., a corresponding pair of noninfected and infected cells from a given batch. However, it should be noted that all conclusions hold valid across all pairs of the respective biological replicates.

### Metabolic Fluxes in Human HFT Cells

Human epithelial cells are the preferred host niche for *C. trachomatis*, which allow optimal bacterial replication and completion of the developmental cycle^15^ (Figure 2A). Phase-contrast images of HFT cells show mature inclusions at 30 h post infection (p.i.) (Figure 2B) and infectivity assays confirm progeny formation, indicative of effective chlamydial replication and development (Figure 2C).

**Figure 2:**
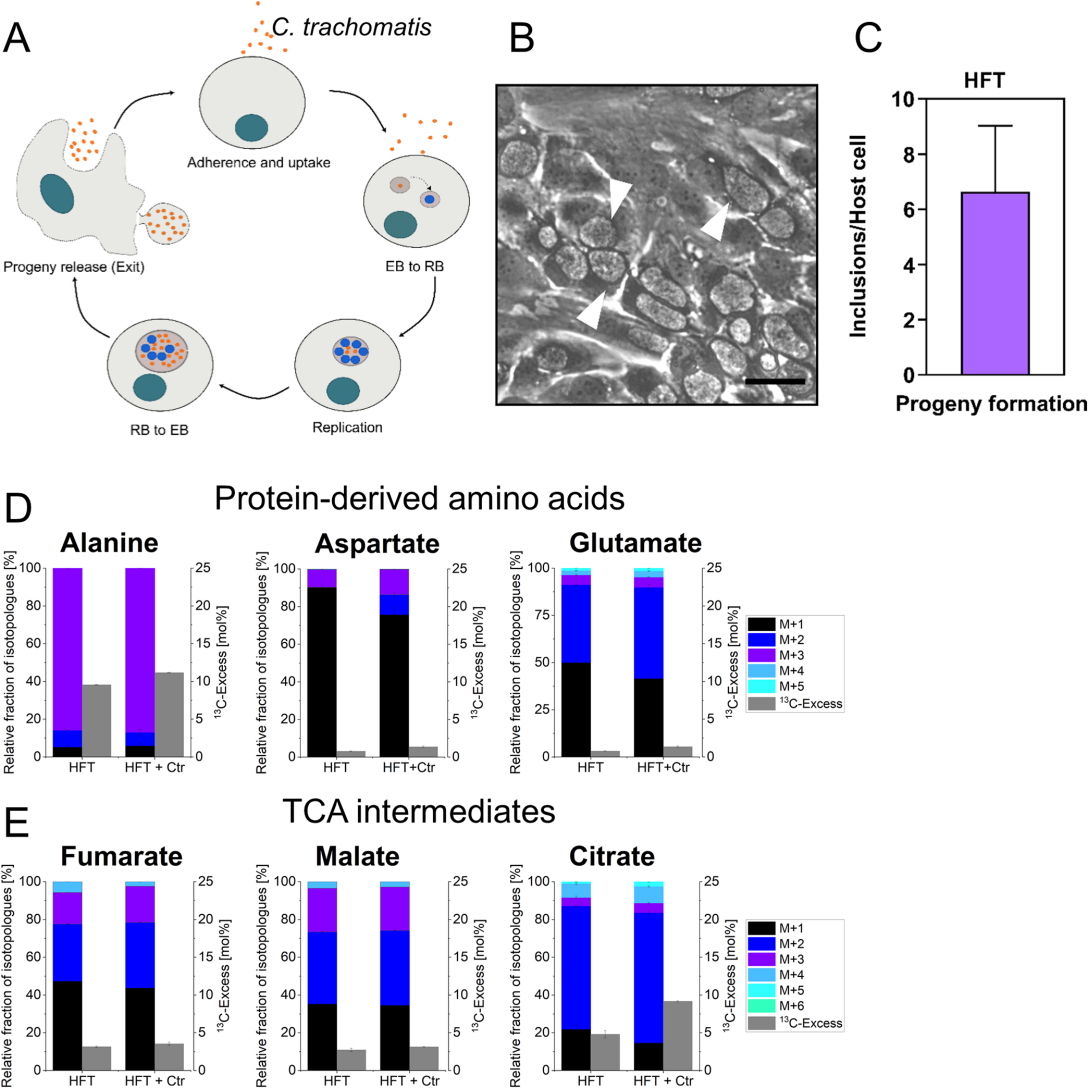
(A) Developmental cycle of *C. trachomatis* in epithelial cells. (B) Mature inclusion formation in HFT cells at 30 h p.i. White arrows indicate *C. trachomatis*-containing inclusions (phase contrast micrographs; scale bar = 20 μm). (C) Infectious progeny quantification (inclusion to host cell ratio) after 30 h of primary infection. Graph shows mean values of independent experiments with + SD (*n* = 3) (EB = elementary body; RB = reticulate body). (D) ^13^C-Excess in mol% and their isotopologue profiles of protein derived amino acids, and (E) TCA cycle intermediates from infected and uninfected HFT cells. M+X represents the molecular mass fraction of protein-derived amino acid specimens carrying X ^13^C-atoms. Error bars show the standard deviations from one biological replicate with three technical replicates; (Ctr = *C. trachomatis*).

### Protein-Derived Amino Acids (Protocol (i))

The essential amino acids leucine, isoleucine, valine, threonine, phenylalanine, histidine, and lysine showed ^13^C-excess values well below 1%. It can be concluded that these amino acids did not acquire ^13^C-label from the supplied [U-^13^C_6_]­glucose and were apparently derived from the unlabeled amino acids present in the RPMI-1640 medium. In contrast, the nonessential protein-derived amino acids alanine, aspartate and glutamate showed a ^13^C-incorporation from the ^13^C-glucose supplement with high ^13^C-excess values for alanine (9.6% in the uninfected HFT; 11.2% in the infected HFT) and lower ^13^C-excess values for aspartate and glutamate (for the uninfected HFT: aspartate 0.8%; glutamate 1.0%; for the infected HFT: aspartate 1.4%; glutamate 1.2%) (Figure 2D). The high fraction of completely ^13^C-labeled alanine demonstrates that this amino acid was made from the fully labeled precursor pyruvate mainly deriving from [U-^13^C_6_]­glucose via glycolysis. The isotopologue profiles of aspartate and glutamate were characterized by M+2 species, which could be explained by metabolic flux via M+2 labeled acetyl-CoA as a precursor for citrate formation in the TCA cycle leading to M+2 labeled 2-ketoglutarate/glutamate and M+2 labeled oxaloacetate/aspartate. The higher ^13^C-enrichment of alanine also reflected that alanine was not present in large amounts in RPMI-1640, whereas both aspartate and glutamate were present in significant amounts. Nevertheless, it can be concluded that glucose was mainly used for host glycolysis and only to a lesser extent to feed into the TCA cycle. The increased ^13^C-excess values especially for alanine from the infected cells clearly indicated that host glycolysis is upregulated due to the infection with *C. trachomatis*. Although alanine biosynthesis is also active in *C. trachomatis*, we conclude that the increase of ^13^C-excess reflected host metabolism because the fraction of bacterial cell mass in the total cell mass of infected HFT cells is only minor (<1%; w/w).

### Intermediates of the TCA Cycle (Protocol (ii))

The cold methanolic extraction afforded intermediates of the TCA cycle. Of special interest was citrate which can only be synthesized by the human host cells since the enzymes isocitrate dehydrogenase, aconitase and citrate synthase are missing in *C. trachomatis*.^5,12^ GC-MS analysis revealed that fumarate, malate, and citrate acquired ^13^C-excess above 2% (Figure 2E). These values increased upon *C. trachomatis* infection. In particular, the ^13^C-excess of citrate was almost doubled. In conclusion, carbon flux into the TCA cycle was increased in response to the bacterial infection. Isotopologue fractions showed mainly M+2 due to citrate synthesis from [U-^13^C_2_]­acetyl-CoA. These profiles were not altered indicating that multiple cycling via the TCA cycle did not increase due to the infection, since this process would have modified the relative distribution of isotopologues with e.g., more M+1 species (Figure 2E).

### Fatty Acids (Protocol (iii))

Following lipid extraction/methanolysis and GC-MS analysis, we observed C16–C24 fatty acids at various relative concentrations (Figure 3A). Notably, the unusual fatty acids heptadecanoic acid (C17:0) and tricosanoic acid (C23:0) were also detected albeit at low amounts. Of special interest were the unsaturated octadecenoic acid (C18:1) and tetracosanoic acid (C24:1) because these were host specific since *C. trachomatis* is not able to form unsaturated fatty acids. The branched-chain fatty acids, iso-C15:0 and anteiso-C15:0, were of chlamydial origin and, in accordance, these fatty acids could only be detected in the infected cells (Figure 3A–D).

**Figure 3:**
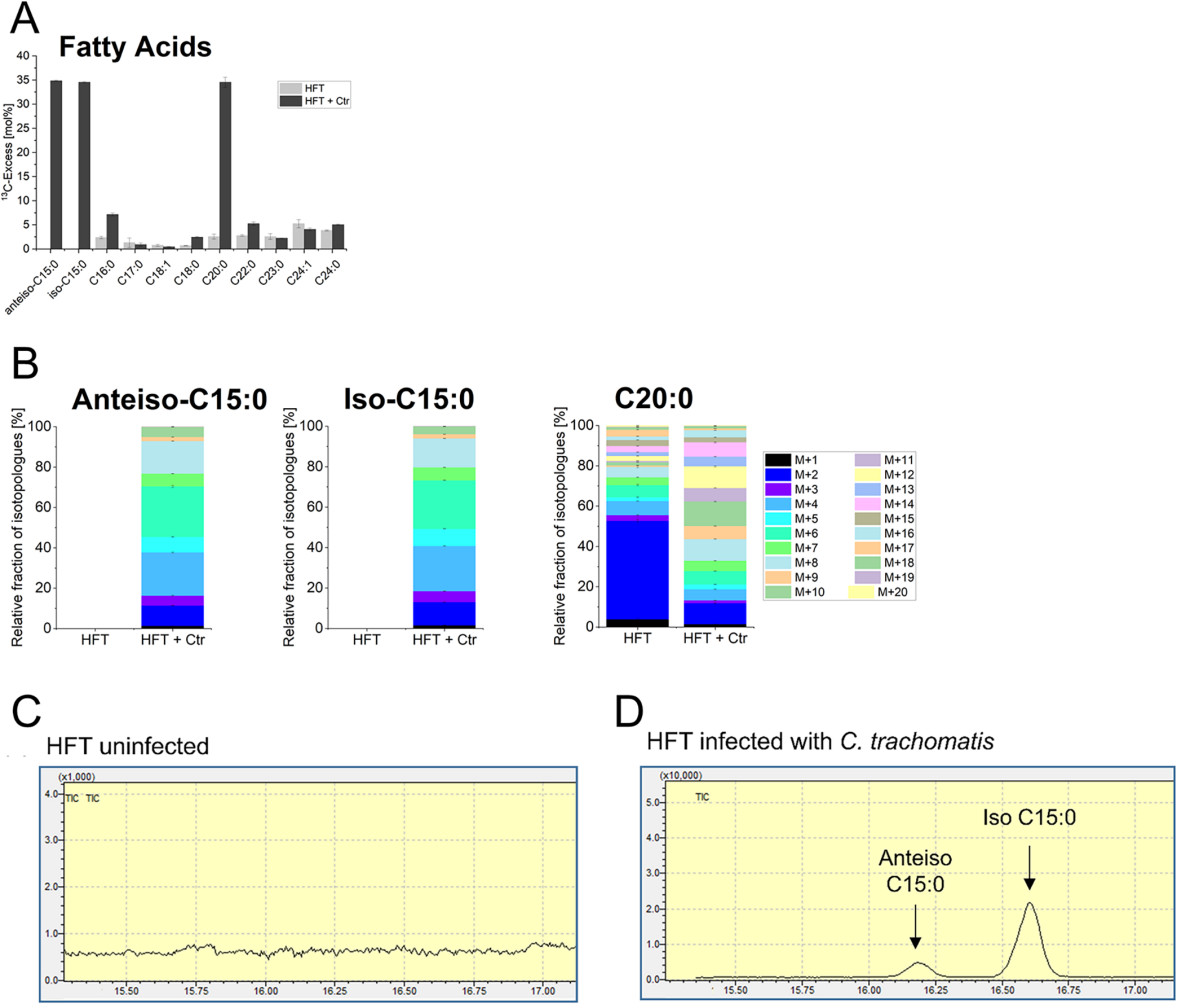
(A) ^13^C-Excess in mol% and (B) isotopologue profiles of selected fatty acids from infected and uninfected HFT cells. M+X represents the molecular mass fraction of fatty acid specimens carrying X ^13^C-atoms. Error bars show the standard deviations from one biological replicate with three technical replicates. (C) and (D) Part of the gas chromatography–mass spectrometry (GC-MS) chromatograms of fatty acids as methyl esters; (Ctr = *C. trachomatis*).

Generally, the ^13^C-excess values of fatty acids obtained from noninfected HFT cells including C18:1 and C24:1 were around 5% reflecting their formation from [U-^13^C_2_]­acetyl-CoA obtained via glycolysis of [U-^13^C_6_]­glucose and conversion of the resulted [U-^13^C_3_]­pyruvate into ^13^CO_2_ and [U-^13^C_2_]­acetyl-CoA. In line with this route and fatty acid biosynthesis based on ^13^C_2_-acetate units, the isotopologue profiles were characterized by M+2, M+4, M+6, etc. using one, two, three etc. units of [U-^13^C_2_]­acetate. Comparison with the ^13^C-excess values and profiles of these fatty acids from infected cells suggested that fatty acid biosynthesis was not upregulated except for arachidic acid (C20:0) which acquired much more ^13^C-label (34.6% from the infected cells compared to 2.6% from the noninfected cells) (Figure 3A). Also, the amount of C20:0 was increased approximately by a factor of 3 in the infected cells (not shown). Not unexpectedly, the ^13^C-profile of C20:0 from the infected cells was also shifted to higher fractions of M+4, M+6, M+8 etc. isotopologues (Figure 3B) in line with the drastically increased overall ^13^C-excess.

The branched-chain fatty acids anteiso-C15:0 and iso-C15:0 with ^13^C-enrichments of approximately 35% could only be detected in infected HFT cells, but not in noninfected HFT cells (Figure 3A). This confirmed that anteiso-C15:0 and iso-C15:0 were indeed produced by *C. trachomatis* by its branched-chain ketoacid dehydrogenase.^26^

The isotopologue profiles of both branched-chain fatty acids displayed a maximum of ten labeled carbon atoms, i.e., the incorporation of five ^13^C_2_-acetate units. The terminal C_5_-moieties of these fatty acids were derived from unlabeled amino acids, i.e., leucine and isoleucine, respectively.

The biosynthesis of anteiso-C15:0 and iso-C15:0 involves 2-methyl-butyryl-CoA (from isoleucine) and 3-methyl-butyryl-CoA (from leucine) as starter units to add five acetyl-CoA moieties. Since the ^13^C-enrichments of leucine and isoleucine were not significant (<1%), we assume that the branched-chain fatty acids were built from unlabeled leucine and isoleucine plus one–five fully labeled acetyl-CoA moieties resulting in M+2–M+10 fatty acids (Figure 3B). Despite its reduced genome, *C. trachomatis* encodes essential genes necessary in fatty acid biosynthesis via the prototypical bacterial type II fatty acid synthase system (FASII), as well as genes encoding phosphatidylethanolamine (PE), phosphatidylglycerol (PG) and cardiolipin (CL) biosynthesis pathways.^5^ Similarly to many bacteria, *C. trachomatis* can synthetize branched-chain fatty acids (BCFAs), with anteiso-C15:0 and iso-C15:0 fatty acids being incorporated into bacterial membrane phospholipids.^10^ Although *C. trachomatis* can hijack host-lipid pathways to acquire host fatty acids,^27^ the bacteria can, in fact, autonomously synthesize lipids required for membranes, without relying on host phospholipids.^26^

The detection of branched-chain C15:0 fatty acids of bacterial origin emphasizes that epithelial cells serve as a stable niche for *C. trachomatis* and allow efficient growth and development of the pathogen.

### Analysis of Supernatants by NMR Spectroscopy

The ^1^H NMR spectrum of the supernatant from uninfected HFT cells (Figure 4A) showed, among others, α-glucose with a characteristic signal for H-1 at 5.24 ppm (i.e., attached to a ^12^C-1) and the ^13^C-coupled satellites at 5.40 ppm and 5.06 ppm (^1^J_CH_, 83.8 Hz) (reflecting H-1 attached to a ^13^C-1) as well as the signal for H-1 of ß-glucose at 4.65 ppm (H-1 attached to ^12^C-1) with the corresponding ^13^C satellite at 4.49 ppm (^1^J_CH_, 82.3 Hz) (H-1 attached to ^13^C-1). The second ^13^C satellite of ß-glucose could not be detected due to overlap with the water signal at 4.80 ppm. Furthermore, a doublet at 1.33 ppm for the methyl group of lactate (^12^C-3 of lactate) with ^13^C satellites at 1.45 ppm and 1.20 ppm (^1^J_CH_, 65.2 Hz) (^13^C-3 of lactate) could be observed (Figure 4B). The ^13^C satellites of glucose and lactate showed higher ^1^H NMR signal intensities in the spectrum of the supernatant from HFT infected cells, whereas other signals were in the same order of magnitude. The fraction of the intensities of the ^13^C-satellites in the global signal intensity of the respective H-atoms (i.e., sum of satellites/(sum of satellites + central signal)) equals the absolute ^13^C-abundances of the respective carbon atoms. The higher ^13^C-enrichment of lactate (74.3%) and the lower amount of glucose (1.3 mg/mL) in the supernatant of infected HFT cells, in contrast to the ^13^C-excess of 68.5% in lactate and 1.4 mg/mL glucose amount in the supernatant of the uninfected HFT cells, implied that more glucose was utilized and glycolysis was increased resulting in the production and secretion of more labeled lactate upon infection.

**Figure 4:**
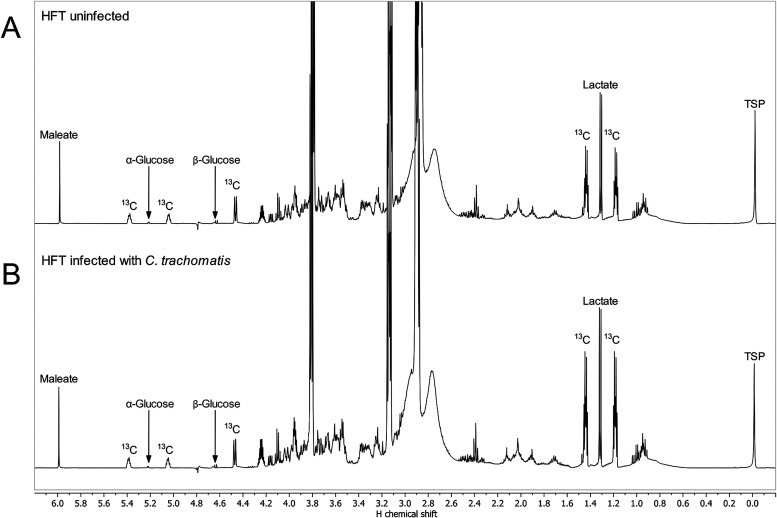
^1^H NMR spectra of supernatants of (A) uninfected HFT and (B) HFT infected with *C. trachomatis*. The residual water signal was suppressed. Labeling was performed with [U-^13^C_6_]­glucose. 1.57 mM maleate was used as an internal standard for the quantification of nuclear magnetic resonance (NMR) signals. 3-(Trimethylsilyl)­propionic-2,2,3,3-d_4_ acid sodium salt (TSP) was used as an internal standard for chemical shift referencing. ^13^C indicate signals due to ^1^H-^13^C coupling.

### Metabolic Fluxes in M1Φ and M2Φ

Immunofluorescence analysis highlights the absence of *Chlamydia-*specific signals in M1Φ (chlamydial protein Hsp60 or the inclusion-specific protein IncA^28^) after 30 h of infection (Figure 5A) and transmission electron microscopy (TEM) analysis confirms absence of chlamydial inclusions (Figure 5B). In addition, recovery of infectious progeny, is a rather rare event, as *C. trachomatis* cannot complete its developmental cycle^17^ (Figure 5C).

**Figure 5:**
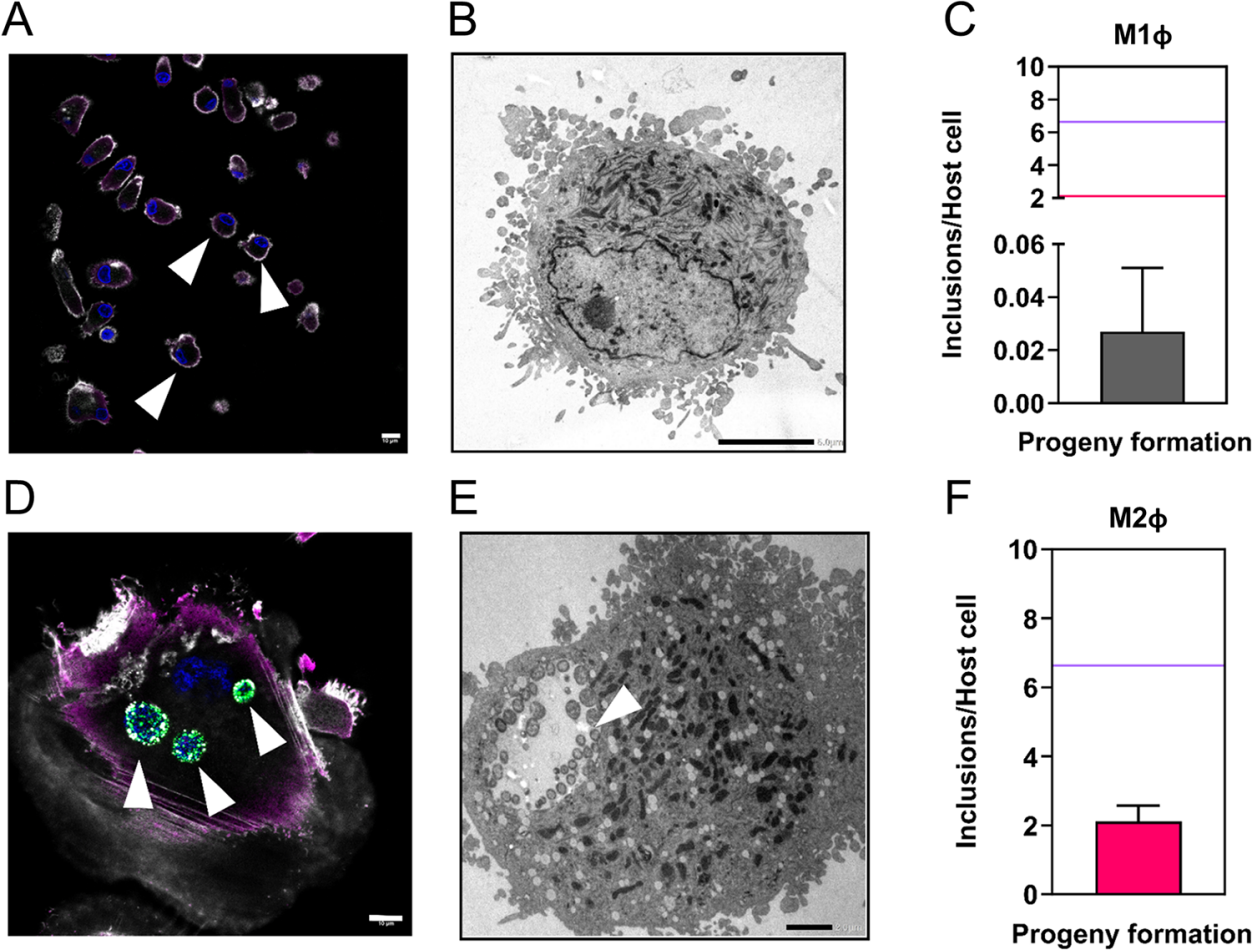
(A) *C. trachomatis*–infected M1Φ. White arrows mark the absence of *Chlamydia*-specific signals (i.e. Hsp60 or IncA) (*gray*: actin filaments; *blue*: Hoechst 34580, nuclei/DNA, scale bar = 10 μm). (B) Transmission electron microscopy micrographs of M1Φ infected with *C. trachomatis* at 30 h p.i. (scale bar = 5 μm). (C) Infectious progeny quantification (inclusion to host cell ratio) after 30 h of primary infection. (D) *C. trachomatis*–infected M2Φ. White arrows indicate mature inclusions *(green*: chlamydial Hsp60; *magenta*: chlamydial IncA, inclusion-specific protein; *gray*: actin filaments; *blue*: Hoechst 34580, nuclei/DNA, scale bar = 10 μm). (E) Transmission electron microscopy micrographs of M2Φ infected with *C. trachomatis* at 30 h p.i. White arrow indicates a *C. trachomatis*-containing inclusion (scale bar = 2 μm). (F) Infectious progeny quantification (inclusion to host cell ratio) after 30 h of primary infection. (C, F) Graphs shows mean values of independent biological replicates with + SD (*n* = 3). Horizontal lines indicate progeny recovery in permissive HFT cells (*purple*) and M2Φ (*pink*), respectively, for comparison.

In contrast, in M2Φ, inclusions can be observed at 30 h p.i., as highlighted by both immunofluorescence microscopy (Figure 5D) and TEM (Figure 5E). Moreover, infectious progeny can be recovered, albeit in lesser amounts than in HFT cells (Figure 5F), thus confirming M2Φ as a pathogen-supporting host niche, in line with previous findings.^17^

M1Φ and M2Φ differ significantly in their antibacterial response. Whereas M1Φ express high levels of antibacterial effectors like inducible nitric oxide synthase (iNOS) or indoleamine-2,3-dioxygenase (IDO), antimicrobial functions are attenuated in unstimulated M2Φ.^19^ Previous studies confirm that M2Φ, but not M1Φ allow development of *Chlamydia* species.^16,17,29,30^

Under stress conditions, such as treatment with IFN-γor penicillin, nutrient deprivation, or coinfection with Herpes viruses, RBs can convert into viable but nondividing, morphologically ARBs, which may reconvert into the infectious EB form when the unfavorable conditions subside.^3,31–33^ Transcriptome and proteome studies have shown that in the persistent ARB state, *C. trachomatis* ceases to produce major structural and membrane components, including lipopolysaccharide,^31^ while synthesis of some proteins, such as the tryptophan synthase (TrpAB) is increased.^3^ Previous studies also indicated that, during infection, both the *Chlamydiae* and host cells undergo specific metabolic changes.^34,35^ However, the precise metabolic fluxes that occur in the RB, the EB, and in particular the ARB states, are largely unknown as are the metabolic conditions of the host cells associated with the different chlamydial states. While we have not observed *C. trachomatis* ARBs in M1Φ, it is likely that the microbicidal conditions encountered within the M1Φ, kill subpopulations of phagocytosed *C. trachomatis* and push the surviving ones toward *persistent*-like states, different from the ARB state. IFN-γ, used to generate M1Φ in our study, is known to induce *C. trachomatis* persistence, both by L-tryptophan depletion (via indoleamine–2,3–dioxygenase, IDO)^36^ and by the STAT1-dependent depletion of the c-Myc transcription factor, and subsequent metabolic rearrangements in the host.^32^ Therefore, survival or selection of quiescent, metabolically inactive *C. trachomatis* subpopulations in M1Φ could explain the absence of bacterial-derived metabolites in this cell type.

### Protein-Derived Amino Acids and Meso-Diaminopimelate (mDAP) Derived from Chlamydial Peptidoglycan (Protocol (i))

Similar to the data from HFT cells, the essential amino acids leucine, isoleucine, valine, threonine, phenylalanine, histidine and lysine were unlabeled in both macrophage subtypes (data not shown). Alanine, aspartate, and glutamate acquired 2–6% ^13^C-excess. The ^13^C-enrichment for alanine was significantly lower in M1Φ (uninfected: 3.2%; infected: 3.3%) and in M2Φ (uninfected: 1.5%; infected: 2.3%) than in the HFT cells (uninfected: 9.6%; infected: 11.2%).

Interestingly, aspartate and glutamate from macrophages showed similar ^13^C-excess values as in the HFT cells. Therefore, macrophages likely utilize significantly less glucose than the HFT cells for glycolysis, whereas a similarly small amount of labeled glucose was fed into the TCA cycle.

No differences in the ^13^C-enrichments and isotopologue profiles of alanine, aspartate, and glutamate could be observed between uninfected and infected M1Φ (Figure 6A), in contrast to the HFT cells (Figure 2D,E) or M2Φ (Figure 6B). Furthermore, we did not detect bacteria-specific metabolites such as the branched-chain C15:0 fatty acids or the bacterial cell wall component meso-diaminopimelate (mDAP) in M1Φ (data not shown). Therefore, we confirm that in M1Φ, no metabolic fluxes between host and bacteria were detected, in line with the notion that M1Φ do not support *Chlamydia* growth.^17,30^

**Figure 6:**
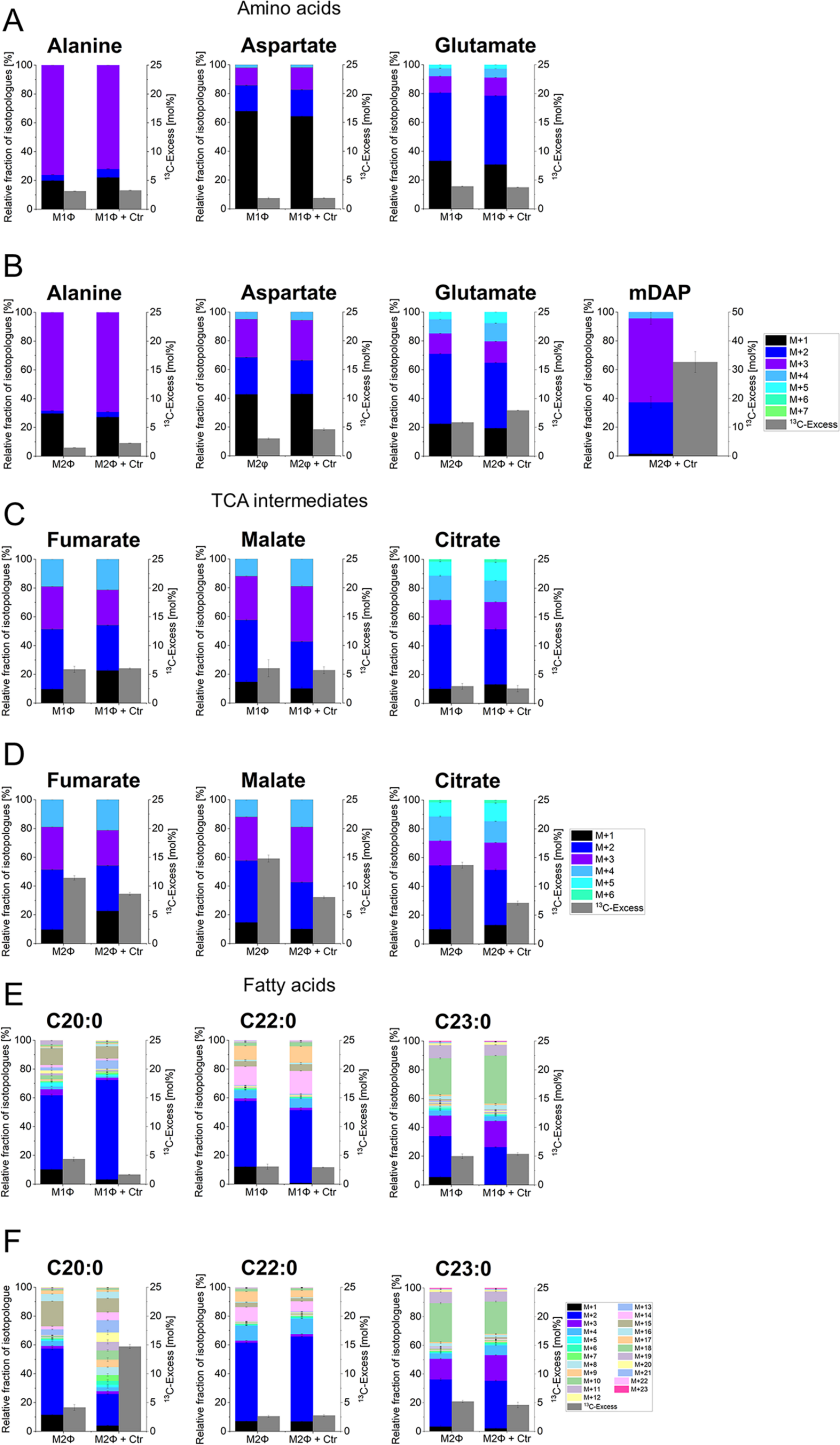
(A, C, E) ^13^C-Excess in mol% of metabolites and their isotopologue profiles from uninfected and infected M1Φ and (B, D, F) from uninfected and infected M2Φ. M+X represents the molecular mass fraction of metabolite specimens carrying X ^13^C-atoms. Error bars show the standard deviations from one biological replicate with three technical replicates; (Ctr = *C. trachomatis*).

Infected M2Φ showed an increase in the ^13^C-enrichment especially for alanine in comparison to the uninfected macrophages, indicating that carbon flux from ^13^C-glucose into pyruvate/alanine via glycolysis was upregulated due to the infection similar as for the infected HFT cells. Accordingly, the isotopologue profiles displayed high fractions of M+3 in alanine.

Interestingly, the bacteria-specific metabolite mDAP was detected with a high ^13^C-excess value of 32.6% (Figure 6B), in contrast to infected HFT cells, where we could not observe mDAP (Figure 2D,E). The isotopologue profile of mDAP revealed that it was synthesized from fully labeled pyruvate leading to M+3 or labeled aspartate mainly leading to M+2 species. Here, the M+3 isotopologues reflected the [U-^13^C_3_]­pyruvate precursor formed by bacterial glycolysis from [U-^13^C_6_]­glucose phosphate. The M+2 isotopologues are explained by the same pattern in aspartate which is related to the ^13^C_2_-unit introduced into the host TCA cycle from [U-^13^C_2_]­acetyl-CoA via ^13^C_2_-succinate, fumarate, malate, oxaloacetate, and finally aspartate. Uptake of aspartate and/or dicarboxylic acids from the host affords the M+2 labeled species in the aspartate semialdehyde precursor for mDAP biosynthesis.

Peptidoglycan is a sugar-amino acid polymer, representing an essential structure in the cell wall of most bacterial species and it is critical for division, osmotic protection, and cell shape maintenance. mDAP is an essential building block of the bacterial peptidoglycan in nearly all Gram-negative bacteria and also a direct precursor of lysine.^37^ In case of *Chlamydia*, the peptidoglycan-cell wall is not assembled in a typical mesh-like sacculus, but solely localized to the septum of actively dividing RBs.^38^

The increase of ^13^C-enrichment in alanine, as well as the detection of the bacteria specific metabolite mDAP in infected M2Φ demonstrated host metabolic adaptation and active bacterial metabolism, as well as bacterial division in these cells (Figure 6B).

### Polar Metabolites (Protocol (ii))

The lower ^13^C-excess values of the TCA intermediates fumarate (uninfected: 5.9%; infected: 6.0%), malate (uninfected: 6.0%; infected: 5.7%) and citrate (uninfected: 3.0%; infected: 2.6%) in M1Φ, indicated that labeled glucose was mainly used for glycolysis and less for the TCA cycle. Again, the ^13^C-enrichments and the isotopologue fractions of these metabolites in uninfected and infected M1Φ were in the same order of magnitude. Therefore, these results verified that *C. trachomatis* infection had no effect on the metabolic fluxes of M1Φ (Figure 6C).

Infected M2Φ showed a decrease in the ^13^C-excess values for the TCA cycle intermediates in contrast to the uninfected host, whereas the isotopologue profiles displayed similar fractions (Figure 6D).

### Fatty Acids (Protocol (iii))

In infected M1Φ, we observed a small decrease in the ^13^C-excess of C20:0 and C24:1, whereas the enrichments of the fatty acids docosanoic acid (C22:0) and C23:0 were in the same order of magnitude. Furthermore, no observable differences in the isotopologue fractions between uninfected and infected M1Φ could be determined (Figure 6E).

In comparison to M1Φ, infected M2Φ showed a marked increase in the ^13^C-enrichment of C20:0 in comparison to the uninfected subtype, similar to infected HFT cells. Moreover, the isotopologue distribution of C20:0 was different between uninfected and infected host cells (Figure 6F). Notably, the bacteria-specific anteiso-C15:0 and iso-C15:0 were not detected in the fatty acids profiles of infected M2Φ.

While the cause of the C20:0 enrichment remains elusive, interestingly, similar observations were made in the case of infection of HeLa cells with intracellular *S. aureus*.^39^ Upon infection, the absolute levels of C20:0 as well as C22:0 and palmitic acid (C16:0) were increased. The authors attributed a cytoprotective role for these fatty acids during bacterial infection, likely resulting from a bacteriostatic effect of the fatty acids against *S. aureus*. The same study^39^ found that coincubation of host cells with heat-killed *S. aureus* also led to an increase in C20:0 production, albeit at a lesser extent than infection with live bacteria. C20:0 biosynthesis might therefore represent a general response to pro-inflammatory signals, such as pathogen associated molecular patterns (e.g. peptidoglycan components, LPS of Gram-negative bacteria, lipoteichoic acid of Gram-positive bacteria).

### Analysis of Supernatants by NMR Spectroscopy

In contrast to uninfected M2Φ (Figure 7A), the ^1^H NMR spectrum of the supernatants of infected M2Φ (Figure 7B) displayed lactate at 1.33 ppm with higher ^13^C satellites at 1.45 ppm and 1.20 ppm, whereas no difference could be detected in the ^1^H NMR spectra of M1Φ (Figure 7C,D).

**Figure 7:**
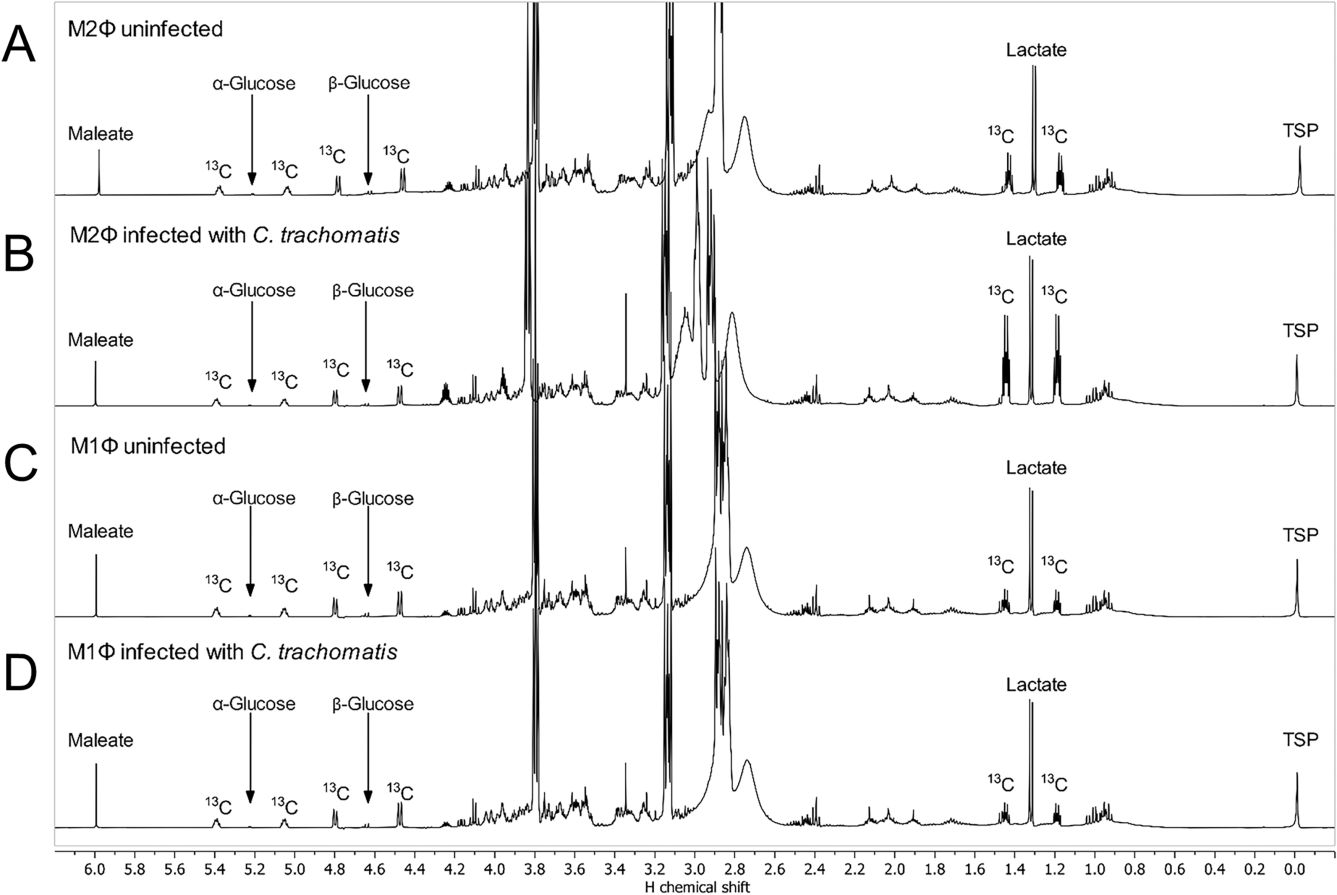
^1^H NMR spectra of supernatants of (A) uninfected M2Φ, (B) M2Φ infected with *C. trachomatis*, (C) uninfected M1Φ, and (D) M1Φ infected with *C. trachomatis*. The residual water signal was suppressed. Sample labeling was performed using [U-^13^C_6_]­glucose. 1.57 mM maleate was used as an internal standard for the quantification of NMR signals. TSP was used as an internal standard for chemical shift referencing. ^13^C indicate signals due to ^1^H-^13^C coupling.

This observation was verified by the calculated ^13^C-excess values of lactate. The enrichment of lactate (72.7%) for infected M2Φ was higher than the lactate enrichment of 60.2% in the uninfected M2Φ. The calculated ^13^C-excess values for the uninfected and infected M1Φ were in the same order of magnitude (uninfected: 51.6%; infected: 51.7%). Furthermore, the glucose amount in the supernatant of infected M2Φ was lower (2.0 mg/mL) than in the supernatant of the uninfected one (2.4 mg/mL). Again, no difference in the glucose concentration could be determined between uninfected and infected M1Φ (both approximately 2.2 mg/mL).

The higher ^13^C-excess value and the lower glucose amount in infected M2Φ indicated that more glucose was used for glycolysis and more lactate was secreted during infection. In contrast, *C. trachomatis* appeared to have no influence on the glucose uptake and lactate production in M1Φ.

### Metabolic Reprogramming of the Host Cells by *C. trachomatis* Infection

A whole genome siRNA screen on the host factors required for chlamydial development revealed that more than 65% of all hits were assigned to host metabolism^40^ underlining the importance of *Chlamydia*–host metabolic interactions.

However, little is known about the metabolic reprogramming of relevant host cells in *C. trachomatis* infections. Most frequently, studies about the metabolic adaptation of host-pathogen interactions were carried out in cell cultures using established cell lines.^41^ Cancer-derived cell lines often exhibit elevated intermediary metabolism characterized by enhanced glucose and (often) glutamine uptake, high (aerobic) glycolysis, increased glucose flux through the pentose-phosphate pathway and (often) enhanced anabolic activities resulting in high rates of amino acid, nucleotide and fatty acid/lipid biosynthesis.^42,43^ These metabolic conditions do not always apply to primary phagocytic immune cells (e.g., monocytes, macrophages, neutrophils), since terminally differentiated cells often exhibit low catabolic and anabolic rates.

Metabolic reprogramming of host cells has been extensively studied in viral infections, rather than intracellular bacterial pathogen (IBP) infections. Here, activation of the host cell metabolism appears to be often achieved by the interaction of specific viral factors with central metabolic regulators, including oncogenes and tumor suppressors, or by the introduction of virus-specific oncogenes.^44^ The few reports on the reprogramming of the metabolism of primary host cells during *ex vivo* and *in vivo* IBP infections suggest that similar mechanisms could also determine the adaptation of the host cell metabolism in IBP infections to provide favorable conditions for intracellular IBP replication.^23,45^

The results of this work are illustrated in Figure 8.

**Figure 8:**
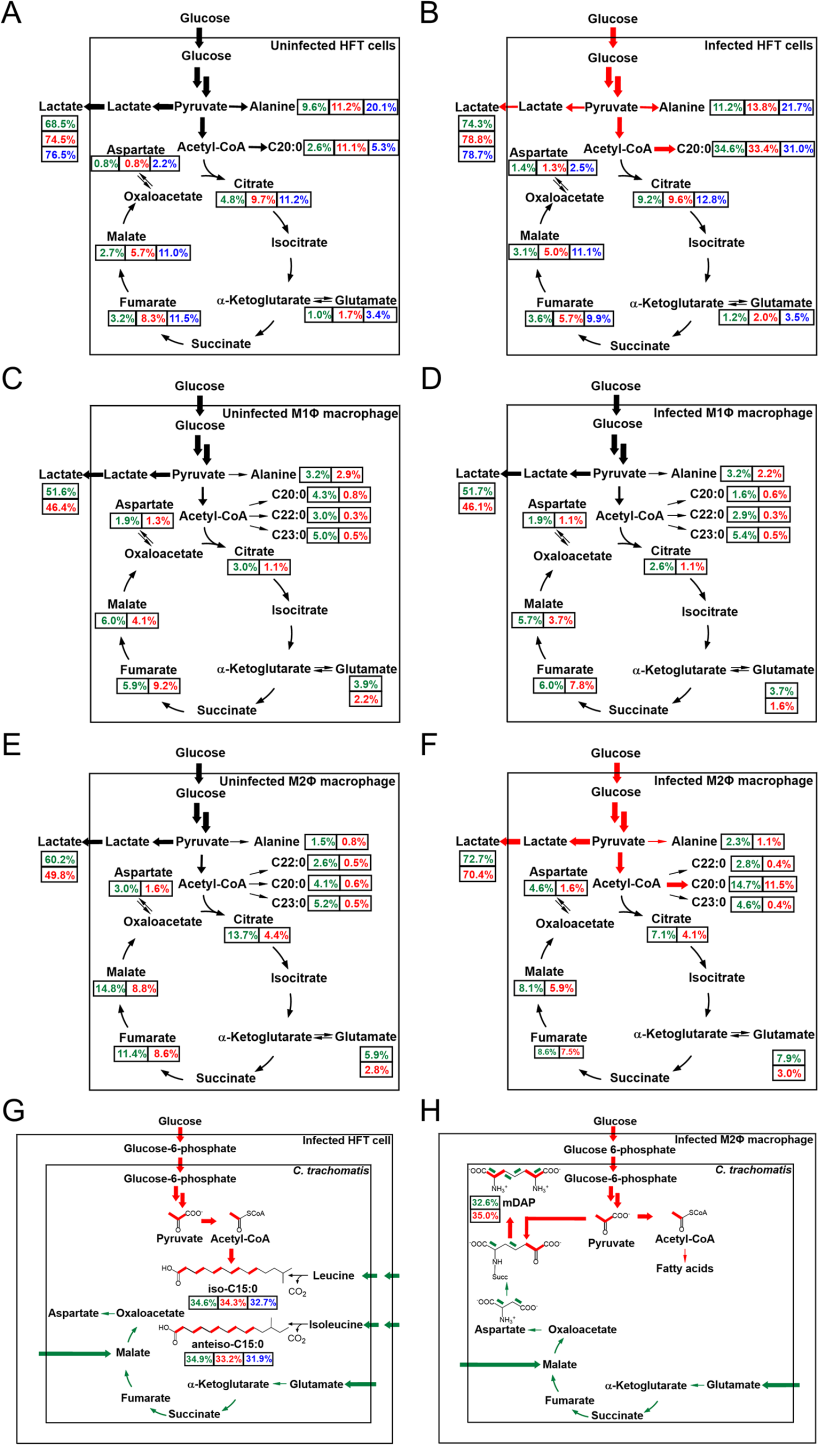
Model for the core carbon metabolism and fluxes of HFT cells and macrophages (M1Φ and M2Φ). The model is based on labeling experiments using [U-^13^C_6_]­glucose as a tracer. The thickness of the arrows reflects the approximate relative amounts of ^13^C-incorporation. The total ^13^C-enrichments from replicate one, two, and three are displayed in green, red, and blue, respectively. (A) Uninfected HFT and (B) HFT infected with *C. trachomatis*. Red arrows indicate the increase in ^13^C enrichments during the infection. (C) Uninfected M1Φ and (D) M1Φ infected with *C. trachomatis*. (E) Uninfected M2Φ and (F) M2Φ infected with *C. trachomatis*. Red arrows indicate the increase in ^13^C enrichments during the infection. (G) *C. trachomatis* in HFT and (H) in M2Φ. Red arrows and segments of structural formulas indicate fluxes and molecular segments based on glucose utilization. Green arrows and green segments of structural formulas indicate fluxes and molecular segments based on amino acid or C4 utilization.

The metabolism of noninfected HFT cells and noninfected macrophages displayed efficient uptake of exogenous glucose and its conversion into pyruvate/lactate by glycolysis. Under our experimental conditions, metabolic fluxes into the TCA cycle and metabolic cycling in the TCA cycle were less efficient (Figure 8A,8C,8E). Together, the cells used for our infection experiments reflected a metabolic state of aerobic glycolysis, i.e. a Warburg-like metabolism. On this basis, enough of the main carbon substrates for intracellularly replicating *C. trachomatis*, i.e., G6P and dicarboxylic acids of the TCA cycle should be available in these host cells, including both M1Φ and M2Φ. Surprisingly, however, the bacteria could only replicate in HFT and M2Φ.

Differential analysis of the labeling patterns, especially of protein-derived alanine, clearly showed that the uptake of exogenous ^13^C-glucose and its conversion into pyruvate/lactate were upregulated upon infection, in HFT cells and M2Φ (Figure 8B,8F), but not in M1Φ (Figure 8D).

The presence of characteristic metabolites illustrates both the ability of *C. trachomatis* to grow and develop in HFT cells and in M2Φ, and its dependence on host cells. The bacterial fatty acids iso-C15:0 and anteiso-C15:0 are partially derived from fully labeled acetyl-CoA (Figure 8G). In infected M2Φ, we detected mDAP which was partially produced from fully labeled pyruvate, made from host-derived G6P via glycolysis. Notably, due to the intrinsic symmetry of mDAP, the profile is randomized, i.e., the M+3 units can be found in both terminal three-carbon moieties of the compound. The same symmetrization can be expected for the ^13^C_2_-units (Figure 8H).

Of note, we measured mDAP production in M2Φ, but not HFT cells. Its absence in HFT cells is, however, not an indicator of halted bacterial replication. *C. trachomatis* establishes inclusions in HFT cells and recovery of infectious progeny at 30 h p.i. demonstrates complete development (Figure 2A–2C). Since peptidoglycan is strictly restricted to the division plane of RBs in replicative form,^46^ one could envision that the timing for sample preparation must match the time-window during bacterial replication, where the likelihood of peptidoglycan enrichment is the highest. At 30 h p.i., bacteria in HFT cells are likely to have already transitioned into EBs, thus mDAP may no longer be detectable. By contrast, in case of M2Φ, a delay in chlamydial development, likely results in late onset mDAP biosynthesis allowing detection at 30 h p.i. However, technical limitations, such as insufficient biological material, cannot be excluded.

Taken together, our isotopologue profiling data reveal how *C. trachomatis* impacts the carbon metabolism of host cells, both cultured (HFT) and primary (human M1Φ and M2Φ). Future approaches employing other stable-isotope tracers could allow additional insight into the changes in other metabolic pathways upon infection with *C. trachomatis*. Experiments using ^15^N-labeled precursors could provide an in-depth analysis of amino acid metabolism, hexosamine metabolism or nucleotide biosynthesis.^47,48^ Glutamine, for example, is required as a nitrogen donor for the *de novo* synthesis of purines and pyrimidines, which serve as nitrogenous bases for nucleosides (i.e., (deoxy)­adenosine/guanosine/uridine/cytidine,5-methyluridine/thymidine). Nucleosides are important metabolites for *C. trachomatis*. The bacteria lack the ability to make purine and pyrimidine nucleotides *de novo*, but they can import ATP and nucleosides from the host.^8,49^ Knowledge of metabolic changes during infection may reveal new targets for drug design and development.

## Methods

### Chemicals

Methanol (MeOH), hydrochloric acid (HCl), glass wool and anhydrous acetonitrile were obtained from VWR (Pennsylvania). Methanolic HCl (3 M) was purchased from Supelco (Pennsylvania). Glass beads were obtained from Carl Roth GmbH & Co. KG (Karlsruhe, Germany). Deuterium oxide (D_2_O), 3-(trimethylsilyl)­propionic-2,2,3,3-d_4_ acid sodium salt (TSP), maleic acid (for synthesis), acetic acid, anhydrous hexane and *N*-*tert*-butyldimethylsilyl-*N*-methyltrifluoroacetamide containing 1% *tert*-butyldimethylchlorosilane (MTBSTFA) were purchased from Merck KGaA (Darmstadt, Germany). Dowex 50WX8 200–400 (H+) was purchased from Alfa Aesar (Karlsruhe, Germany). Distilled water was obtained from an in-house purity water system.

### Cell Lines and Bacteria

Human immortalized fallopian tube secretory epithelial cells (FT190, henceforth referred to as HFT),^50^ and primary human macrophages were used for all infection experiments. HeLa 229 cells (ATCC CCL-2.1) were routinely used as growth substrate for *Chlamydia.* Unless stated otherwise, both infected and unifected cells were cultured in RPMI-1640 (Thermo Fisher Scientific, #72400054) supplemented with heat-inactivated (56 °C, 30 min) fetal bovine serum (FBS 10%; v/v), in a humidified atmosphere, with 5% CO_2_ at 37 °C and routinely tested for *Mycoplasma* contamination by PCR.

In this study, *C. trachomatis* serovar L2/434/Bu (ATCC VR-902B) were used. Bacteria were propagated on HeLa229 cells and purified as described^30^ by Gastrografin (Bayer, #86971488) gradient ultracentrifugation (20 and 50%; v/v).

### Isolation of Primary Human Monocytes and Differentiation to Macrophages

Primary human macrophages were derived from peripheral blood mononuclear cells (PBMCs) isolated from leukoreduction system cones as described.^30^

### Infectivity Assay

Host cells (macrophages or HFT) were seeded in 24-well plates at a density of 10^5^ cells/well, and infected with *C. trachomatis* at MOI of 1. After 2 h of cocultivation, extracellular bacteria were removed by washing twice with DBPS (Thermo Fisher Scientific, #14190169), followed by addition of fresh medium, and further incubation (*primary infection*). At 30 h p.i., cells were lysed with glass beads, and lysates were used to infect fresh HeLa229 cells (*progeny formation*) for 18 h. Inclusions and host cells were manually counted, and *progeny* formation was expressed as *inclusion to host cell* ratio. If no inclusions were visible after 24 h, infection was prolonged for a maximum of 50 h before analysis.

### Immunofluorescence Analysis and Electron Microscopy

Host cells were seeded onto cover slides and infected with *C. trachomatis* at MOI of 1 for 30 h as described above. Samples were subsequently washed with DPBS and fixed with 4% PFA (Morfisto, # 11762.01000) for 30 min at RT. Fixed cells were permeabilized with 0.2% Triton-X-100 in DPBS for 15 min. Sample blocking, and antibody or stain dilutions were done in 2% FBS in DPBS (v/v) for 1 h, at RT, each. The following primary antibodies were used: chlamydial HSP60 (Santa Cruz, mouse, #sc-57840, 1:200) and polyclonal serum against IncA (custom-made, Gramsch laboratories, rabbit, 1:100). The following secondary antibodies were used: anti-mouse IgG Alexa Fluor488 (Thermo Fisher Scientific, #A-11001, 1:300) and anti-rabbit IgG CF568 (Sigma Aldrich, # SAB4600401, 1:100). Actin filaments were stained with Phalloidin iFluor-647 (Abcam, # ab176759, 1:100) and nuclei were stained with Hoechst 34580 (Thermo Fisher Scientific, # H21486). Coverslips were mounted using Mowiol, air-dried for 24 h and imaged on a Leica TCS SP5 confocal microscope using a 63x oil immersion UV objective with a numerical aperture of 1.4. Image processing was performed using Fiji.^51^

For TEM analysis, cells were infected as described above. At 30 h p.i., samples were fixed and processed as described.^6^

### Infection and Sample Preparation for Isotopologue Profiling

HFT cells and human M1Φ and M2Φ were infected with *C. trachomatis* at MOI of 1 for 2 h to allow bacterial uptake by host cells. A total of 6 × 10^6^ and 3 × 10^6^ cells were used for infected and uninfected samples, respectively. At 2 h p.i., cells were washed once in DPBS and medium was exchanged to glucose-free RPMI-1640 (Thermo Fisher Scientific, #11879-020) supplemented with HEPES (25 mM), FBS (10%; v/v) and either [U-^13^C_6_]­glucose (2 g/l; 11.11 mM) (labeled samples) or d­(+)-glucose (unlabeled). At 30 h p.i., plates containing cells were washed with cold DPBS and snap frozen in liquid N_2_. Supernatants were also collected, sterile-filtered with 0.2 μm-pore filters and snap-frozen. Plates were then thawed on ice and samples were collected by scraping with ice-cold methanol/H_2_O (80/20; v/v). Samples were dried under a gentle stream of nitrogen gas, flash-frozen in liquid N_2_, and lyophilized overnight.

### Analysis of Polar Metabolites

The analysis was done as described.^52^ In brief, a part of the dried cell pellet was dissolved in 1 mL cold MeOH. 800 mg of glass beads were added. Cells were lysed in a ribolyser system (1 × 20 s at 4.5 m/s; 2 × 20 s at 6 m/s). Samples were centrifuged for 10 min at 4900x *g* and supernatants were dried under a gentle stream of nitrogen gas. Derivatization was performed with 25 μL acetonitrile (anhydrous) and 25 μL MTBSTFA for 1 h at 70 °C.

### Analysis of Protein-Derived Amino Acids and mDAP

Analysis was performed as described.^53^ In brief, a part of the dried cell pellet was hydrolyzed with 500 μL HCl (6 M) at 105 °C overnight. The solution was dried under a gentle stream of nitrogen gas and the residue was dissolved in 200 μL acetic acid (50%). For purification, a cation exchange column of Dowex 50WX8 (H+ form; 7 by 10 mm; 200–400 mesh, 34 to 74 μm) was prepared which was previously washed with 1 mL MeOH (70%) and 1 mL distilled water. After addition of the dissolved amino acid solution, the column was washed twice with 800 μL distilled water. For elution of the amino acids, 1 mL ammonium hydroxide (4 M) was used. The eluates were dried under a gentle stream of nitrogen gas. The derivatization was done with 25 μL acetonitrile (anhydrous) and 25 μL MTBSTFA for 30 min at 70 °C.

### Analysis of Fatty Acid Methyl Ester

Analysis was performed as described.^54^ In brief, a part of the dried cell pellet was derivatized with 0.5 mL methanolic HCl (3 N) at 80 °C overnight. The solution was dried under a gentle stream of nitrogen and dissolved in 50 μL hexane (anhydrous).

### Analysis of Supernatants

A volume of 1 mL of the supernatant was lyophilized overnight. The dry residue was dissolved in 50 μL TSP solution (c = 2 mg/mL), 100 μL maleic acid solution (c = 1 mg/mL) and 450 μL D_2_O. 550 μL of the solution was transferred to a 5-mm NMR tube.

### Gas Chromatography–Mass Spectrometry (GC-MS) Measurement Parameters

All GC-MS spectra were recorded on a QP2010 Plus gas chromatograph mass spectrometer (Shimadzu) which was equipped with a fused silica capillary column (30 m × 0.25 mm; 0.25 μm film thickness, SUPELCO) and a quadrupole detector (with electron impact ionization at 70 eV). The derivatized samples were measured three times (technical replications) in selected ion monitoring (rate 0.5 s) at an interface temperature of 260 °C and a helium inlet pressure of 70 kPa in split mode (1:5). For the analysis of polar metabolites, the column was heated to 100 °C and held for 2 min. Then, the following temperature gradients were used: the first temperature gradient was 3 °C/min to a final temperature of 234 °C, the second gradient was 1 °C/min to a final temperature of 237 °C, the third gradient was 3 °C/min to a final temperature of 260 °C and the forth gradient was 10 °C/min to a final temperature of 300 °C, which was held for 2 min. For the analysis of protein-derived amino acids and mDAP, the column was heated to 150 °C and held for 3 min. Then, a temperature gradient of 7 °C/min was used to a final temperature of 280 °C which was held for 3 min. For the analysis of fatty acids, the column was heated to 150 °C and held for 3 min. Then, the following temperature gradients were used: the first gradient was 1 °C/min to a final temperature of 187 °C, the second gradient was 2 °C/min to a final temperature of 220 °C and the third gradient was 10 °C/min to a final temperature of 300 °C, which was held for 2 min. Data were processed with LabSolution Version 4.11 (Shimdazu, Duisburg, Germany). The calculation of ^13^C enrichment and distribution of isotopologues was done as previously described.^55^

### NMR Measurement Parameters

All NMR spectra were recorded with a Bruker AVANCE III 500 MHz spectrometer equipped with a SEI probe using TopSpin Version 2.1 (Bruker Biospin GmbH, Rheinstetten, Germany). Prior to the NMR measurement, deuterated water was added to a final concentration of 10% (v/v). Supernatants of HFT cell cultures were measured at 294 K, supernatants of macrophage cell cultures were measured at 298 K. The pH values of the NMR samples were 7.2–7.4. ^1^H NMR spectra were recorded with the Bruker pulse program “noesygppr1d” for suppression of the water signal during the relaxation period applying a narrow saturation pulse with a line width of about 25 Hz. The parameters were ns = 64, ds = 4, aq = 5.45 s, td = 65536, si = 65536, sw = 12.0 ppm, p1 = 8.20 μs. Prior to Fourier-transformation, the FIDs were multiplied with a mild Gaussian function (lb = −0.10; gb = 1.66). Chemical shifts were reported relative to the internal standard TSP at 0 ppm. Maleic acid was used as internal standard for the quantification of glucose amount. Data were processed with MestreNova Version 12.0.0 (Mestrelab Research, Santiago de Compostela, Spain).
